# When Shall the Uterus Be Removed in Pelvic and Abdominal Surgery?1Read at the thirtieth annual meeting of the Medical Association of Central New York, held at Buffalo, October 19, 1897.

**Published:** 1898-05

**Authors:** C. C. Frederick

**Affiliations:** Buffalo, N. Y., Surgeon-in-chief Buffalo Woman’s Hospital.


### WHEN SHALL THE UTERUS BE REMOVED IN PELVIC AND ABDOMINAL SURGERY?[1]

By C. C. FREDERICK, B. S., M. D., Buffalo, N. Y.,
Surgeon-in-chief Buffalo Woman's Hospital.

THE object of this paper is to help, so far as it can, to further a spirit of conservatism in pelvic and abdominal surgery. I wish to be understood as condemning all operations upon women which are not positively indicated. Too many women in the past have been the subject of operative procedures, when general treatment for general neurasthenia would have been more proper and much less harmful. I believe that true conservatism consists in operating only when an operation is indicated, to do all that is necessary at that time, provided the patient's condition will allow it, and to leave her in as nearly a normal condition after operation as possible. I know from my own observations in many operating rooms that in the past and at present many women are subjected to the removal of normal organs, which ought to be preserved. Thousands of women have had tubes, ovaries and wombs removed early in their child-bearing period, whose lives would have been far happier and whose health would have been far better if it had not been done.

Woman was created with pelvic organs which were designed to be in functional activity during a period of about thirty to thirty-five years. If these functions are artificially stopped prior to the period of their normal cessation, it is reasonable to suppose that the woman's condition will not be so good as when she is having the normal functions. Such are the facts of experience. The further away from the age of the normal menopause we produce an artificial menopause, the more profound are the nervous disturbances which result. So many operators seem to forget that the pelvic organs were placed there for a specific purpose and that we ought, conscientiously, to preserve every normal structure which is found. A great deal has been written about the ill results of leaving the uterus in the pelvis. Very little has been written, however, about the ill results of the

1. Read at the thirtieth annual meeting of the Medical Association of Central New York, held at Buffalo, October 19, 1897.

indiscriminate removal of these organs when they were not totally diseased. The French school of vaginal hysterectomists and their followers everywhere have done much of this, to me, unjustifiable sacrifice of normal organs. That is the reason why I am so much opposed to vaginal pelvic surgery. Many a uterus and many a tube and ovary are removed by this route, when by the abdominal route, if the operator were conservatively inclined, the organs might be preserved, simply because they could be handled and inspected thoroughly before any steps are taken which necessitate their removal. I am not speaking now from prejudice; I am speaking of what I have positively seen done by prominent operators in this country. When I have witnessed these operations, I have thought, if it be true that the truth must prevail, this method of operating would soon be relegated to its proper place in surgery and not be carried to an extreme, as it has been, entirely detrimental to the physical well-being of the suffering women who seek relief at our hands.

It has been asserted by some operators that the uterus itself was often a source of distress if left in the pelvis after removal of a part of the adnexa for chronic inflammatory disease; therefore the belief arose that if the uterus and the other healthy adnexa were to be removed that the patient would be better. I have tried to keep informed, so far as it is possible, of the results of operations upon every patient. In comparing these results I cannot ascertain that those from whom I have removed the uterus, tubes and ovaries for chronic pelvic inflammations have progressed any more rapidly, or have recovered any better degree of health than have those in whom I have preserved the uterus and perhaps only a small piece of one ovary. I have noticed that for one or two years those from whom I have removed all the pelvic organs have had exaggerated nervous disturbances incident to the menopause and many of them have been wretched. Some have not suffered so much. Many of them at first have had a return of sexual desire which has gradually waned and eventually been lost. On the contrary, those whose uteri and an ovary or a part of it, ever so small, has been left, the menstrual function has returned, sexual appetite has become normal and remained so. It not infrequently happens that a menorrhagia or too much uterine discharge may necessitate a second curettage for its relief, but that is nothing compared to the fact of being totally unsexed, or to the despondency which I find harasses these unfortunates who are no longer wives to their husbands.

The case must be indeed a bad one of chronic inflammation of the appendages in which no part of either ovary is not found sufficiently normal to resect and leave in situ. It is now nearly two years since I have removed the uterus in any case of this kind, because I have diligently sought for a chance to leave it. In seeking for that chance I have also sought diligently to find a small part of one or both ovaries which I could resect and leave in. I have never had occasion to regret the effort expended in this direction. I have also endeavored to be conservative toward the tubes also. I am of the opinion that if both tubes and ovaries must be removed entirely it is as well or better to remove the uterus also by supravaginal amputation, which is easier than total extirpation. The uterus after removal of both adnexa is a useless organ and it may be a source of subsequent danger by the development in it of new growths, benign or malignant in nature. The operation is much simplified by removing the uterus, leaving in the stump of the cervix. A case so bad as to necessitate the removal of all of both adnexa will have large oozing and bleeding surfaces after they have been torn from their dense beds of adhesions. These denuded spots are removed with the uterus and instead of raw denuded and oozing points on the peritoneum we substitute a clean peritoneum by sewing the flaps of peritoneum over the cervical stump. The operation is shorter, the hemostasis is more perfect and the peritoneum is left healthier and cleaner. Therefore, from the standpoint of convenience and expedition I rather remove the uterus than do a conservative operation, where one has to tear up adhesions, sew over oozing points and resect and sew up tubes and ovaries. It takes longer to do conservative work and requires much more skill.

I have an experience in two cases from whom I removed double ovarian cysts at about the period of the normal menopause. One occurred in 1895 and the other the present year. Both were feeble women, the uterus was apparently perfectly normal and as I desired to make the operation as short as possible I removed the double cyst and closed the abdomen. Both developed carcinoma of the uterus within six months and when they returned to me for second operation were too far advanced to do a hysterectomy. These are not the only old women from whom I have removed both ovaries and not the uterus. But these two developed cancer, which might have been obviated had I removed the uterus at the time of operation. I believe it wise to remove the uterus always in women at, near, or past, the menopause if it can with safety be done during the course of operation.

Until recently the operation for fibroid tumors of the uterus has been total extirpation. There is a class of soft, bleeding fibroids that can be relieved and sometimes cured by tying the uterine arteries through the vagina. In 1895 I reported to the American Medical Association my experience in five cases. This plan is not applicable to all fibroids and if used will not benefit any but the one class—namely, small, soft, bleeding fibroids.

Within the past three years I have enucleated from the uterus intra-abdominally multiple fibroids of the uterus in about a dozen cases, leaving the uterus in situ. As many as fourteen fibroids have been removed from one uterus, ranging in size from a marble to a large orange, some sessile, others partly pediculated. These have all been in young women under forty, most of them about thirty, anxious to bear children. I do not think that hysterectomy is a profitable operation in these cases. The same facts hold true of this conservative operation as do in chronic pelvic inflammations. It is much easier to do the hysterectomy. It is often difficult to control oozing from the uterine walls. Dr. Howard Kelly, of Johns Hopkins, I notice, has just published a paper on this subject in the *Journal of the American Medical Association.* He also now is advocating more conservatism than formerly in the treatment of multiple fibromata. The necessity for hysterectomy in the treatment of fibroids is now confined to the hard interstitial myo-fibromata.

And now I come to the consideration of hysterectomy for malignant growths of the uterus. When should the uterus be removed for malignant growths? If the malignancy has gone beyond the limits of the uterus, it is useless to subject a woman to the pain and danger of hysterectomy, for it will certainly recur. She may live longer even when it does recur, but every aspect of the disease is the same except that the uterine hemorrhages are absent in its fatal course. I have long since ceased doing hysterectomies unless I can positively find, under anesthesia, no invasion of the tissues adjacent to the uterus.

Right here I wish to drop a suggestion to gentlemen in family practice as to the early diagnosis of malignant disease of the uterus. There is not one case out of five which I see with uterine cancer that is not too far advanced to give the woman any chance of cure by hysterectomy. Uterine hemorrhage between the ages of thirty-five and fifty is too frequently carelessly taken to be due to the menopause. Uterine hemorrhage is always a danger signal, a red flag thrown out as a warning. If these cases are examined early and diagnoses made, hysterectomy promises well for them. The trouble is, and always has been, that most of the cases upon which we do do hysterectomy for cancer are too far advanced—most of them have a history of hemorrhage for from six months to one year. Hysterectomy could be done early, return of the disease could be prevented and the large mortality from uterine cancer could be very materially lessened by early diagnosis of malignancy.

64 Richmond Avenue.

